# A Case of Post-Diphtheritic Paralysis

**Published:** 1876-08

**Authors:** A. F. Reed

**Affiliations:** Harv.


A Case of Post-Diphtheritic Paralysis—*By A. F. Reed, M.D. Harv.*—In October, 1875, being twenty-six years of age and in good health, after two months' constant exposure to diphtheria, I was inoculated from a child two years old, who, on examination, coughed portions of the membrane into my face. Six days after this exposure I was seized with a chill, followed the next day (October 28th) by the appearance of a diphtheritic deposit on one tonsil. The deposit was limited to the tonsils and back part of the pharynx, and in nine days disappeared. Exhaustion and great gastric irritability retarded convalescence. Four weeks passed before I was able to sit up. Two weeks after convalescence was declared, a sharp, lancinating pain in the left axilla was noticed, recurring two or three times at short intervals. In a few days, after seeing visitors or talking a little, severe and constant pain in the elbow-joints occurred, which soon extended to the muscles of the arm and chest. After resting these pains diminished or disappeared, and in a week entirely ceased. On attempting to rise, my limbs seemed surprisingly weak, but at the expiration of the sixth week a short walk was possible. After a brief period of improvement, my legs began to grow uncertain and weak, and by December 10th I could take but a few steps. At this time a partial loss of sensation came on, beginning in the feet and gradually progressing to the trunk, together with a feeling of coldness in the feet, which, however, were not cold to the touch. This numbness increased faster than the loss of motion. Soon after its appearance in the lower extremities, the ends of the fingers lost their sense of touch; the loss of power also extending in a week to the elbows, and at no time greatly affecting the arm. Loss of motion in the fingers and fore-arm accompanied it and increased for some weeks. The mouth, tongue, and portions of the face lost their sensitiveness at the same time and to the same degree. In a few days my voice grew thick, and was soon like that caused by cleft palate. The soft palate and uvula hung loosely in the mouth, and on attempting to swallow fluids they were regurgitated through the nares. Dimness of vision for a short time prevented reading. In three weeks my voice, then at times unintelligible, grew suddenly better, and in four or five days was restored. The difficulty in swallowing also soon disappeared. The loss of motion and sensation in both arms and legs increased. In walking I seemed to be on velvet; there was a sensation of coldness in my feet, and at first the circulation was retarded. The general loss of power was progressive until February 1st. It was then impossible for me to stand alone even when lifted up, to raise myself an inch from the chair by my arm, to bring my thumb and fore-finger together, or to exercise any strength in any part. The toes hung lifeless, and no reflex action was produced on tickling the sole of the foot. The urine was voided with difficulty, and the power of erection was gone. The interosseous muscles were wholly paralyzed, though still reacting to the faradic current. The fingers were drawn up when the hand was at rest, but only by great effort could be straightened out again. The muscles of the arm were much weakened, but with those of the thigh retained more power than the rest. They were also the last to lose and the first to gain motion. All these muscles were more or less responsive to the faradic current, the gastrocnemius least of all. During the weeks previous and at this date my appetite was excellent, and my food well digested. From this time an improvement as gradual as the invasion was noticed. In one week I could lift my body in the chair an inch or two, and when standing felt more secure. In two weeks I could raise myself up from the chair mainly by my arms, and undressed without aid. At the end of three weeks I could walk about the room aided by a cane, and wrote legibly. The difficulty in voiding the urine and loss of power of erection had by this time gone. In four weeks I walked out for a short distance, and in two weeks more all paralysis had disappeared, leaving some neuralgic pains in the knees and feet, which lasted but a short time. On April 1st I walked several miles without great fatigue. Atmospheric changes made no change in my strength. Insomnia was the greatest annoyance suffered while confined to the house. Three or four hours' sleep were all that could be obtained. The loss of sleep did not, however, leave me unrefreshed.

*Treatment.*—From January 12th, faradism to the muscles every day until February 15th; afterwards three times a week for three weeks. Tincture of nux vomica and tincture of phosphoric ether were given for ten days. The stomach rejecting these, one-thirtieth of a grain of strychnine was substituted, which was increased to one-fifteenth three times daily for six weeks. A pint of ale daily for two months. Friction and kneading of muscles every morning for one hour.—*Boston Medical and Surgical Journal.*

